# Temporal and Spatial Variation Characteristics of Water Quality in the Middle and Lower Reaches of the Lijiang River, China and Their Responses to Environmental Factors

**DOI:** 10.3390/ijerph19138089

**Published:** 2022-07-01

**Authors:** Dantong Zhu, Xiangju Cheng, Wuhua Li, Fujun Niu, Jianhui Wen

**Affiliations:** 1 State Key Laboratory of Subtropical Building Science, South China University of Technology, Guangzhou 510640, China; 20209033@scut.edu.cn (D.Z.); niufj@scut.edu.cn (F.N.); 2 School of Civil Engineering and Transportation, South China University of Technology, Guangzhou 510640, China; ctliwuhua@mail.scut.edu.cn; 3 South China Institution of Geotechnical Engineering, School of Civil Engineering and Transportation, South China University of Technology, Guangzhou 510640, China; 4 Guilin Environmental Monitoring Center, Guilin 541002, China; midtrek@163.com

**Keywords:** environmental factors, water quality indicators, temporal and spatial distribution, Lijiang River Basin, sensitivity, contribution rate

## Abstract

As the climate and the external environment have changed, the environmental factors of the Lijiang River Basin (LRB) have changed, posing new threats to the environmental quality, ecosystem balance, and management and protection of the water environment of the Lijiang River. Water quality indicators and environmental factors vary spatially along the Lijiang River, which runs through urban areas, farmland, and karst areas. However, research on the response of water quality to water environmental factors is still lacking. Within this context, this study considered statistical methods and hydrological, meteorological, and water quality data of the middle and lower reaches of the Lijiang River from 2012 to 2018, expounded on the temporal and spatial change characteristics and evolution trends of water quality indicators; we analyzed the correlation between water quality indicators and environmental factors; we quantitatively assessed the sensitivity and contribution rate of water quality indicators to environmental factors. The results demonstrated that rainfall feedback on the river streamflow was lagging, and upstream precipitation often affected downstream streamflow. The water quality in the upper reaches of Guilin has improved year by year, and pollution levels have increased slightly when flowing through the urban area of Guilin. In spite of this, it still falls within the range of self-purification. River characteristics heavily influence the impact of environmental factors on water quality indicators; in contrast, the effects of different locations along the same river are more similar. Four water quality indicators are negatively correlated with water temperature, pH, and dissolved oxygen (DO). The sensitivities of ammonia nitrogen (NH_4_-N) and chemical oxygen demand (COD_Mn_) to streamflow increase with the flow direction. The contribution rates of DO-to-total phosphorus (TP) and pH-to-TP are over −6%. Water temperature is the major contributing factor in the Lijiang River, while DO has a higher contribution in tributaries. The external sources affect the concentration of various water quality indicators and the sensitivity of water quality indicators to the external environment. There should be a series of measures implemented to reduce pollution, such as using oxygenation or chemical means to increase pH in Dahe and Yangshuo to control water pollutants. Tourism and particular karst topography make LRB’s calculations unique, but the research method can be applied to other watersheds as well.

## 1. Introduction

The Lijiang River is an essential part of Guilin’s celebrated scenery. It was one of China’s 13 nationally protected rivers in 1978. The Lijiang River has multiple functions, including water supply, irrigation, fishery, storage, and navigation, and its ecological environment quality is closely related to people’s health and social economy. In recent years, with rapid economic development, population growth, and the vigorous development of tourism, land use patterns have undergone tremendous changes [1,2], and a large amount of domestic sewage and agricultural wastewater have entered water bodies, seriously threatening water quality [3]. As the mother river of the Guilin region, the water volume of the Lijiang River is small in the dry season, the capacity of river’s dilution and self-purification is poor, and given the discharge of agricultural sewage and domestic sewage, the water environment quality of Lijiang River is not optimistic. In June 2014, Guilin Karst was selected as an important nominated site for “the second phase of karst in South China” and was successfully listed as a World Natural Heritage site. How to protect the water environment quality of the Lijiang River Basin (LRB) is particularly important for the preservation of human health and the landscape in the region.

Water resources in the Lijiang River vary dramatically, and the quality of the water environment has indicated significant temporal and spatial differences [4,5]; meanwhile, as a karst area, there are many underground rivers in the LRB, and the water in karst areas generally runs off fast through conduits to springs [6], due to these unique hydrogeological features, karst systems are particularly vulnerable to contaminants [7]; these pose challenges for the control and prevention of water pollution in the Lijiang River. The Lijiang River is a rain-source river in the monsoon area. With monsoon climatic conditions occurring during the same season as rain and heat, the change in runoff is closely related to the temporal and spatial distribution of precipitation [8,9]. Ecosystem health refers to an ecosystem in a region being in a good balance, therefore maintaining the integrity of the natural ecosystem, economic system, and social system; it can provide tourism and production and life services for tourists and local residents; and it has the ability to self-adjust and repair external disturbance [10,11]. With the destruction of the water-source forest community structure and the increase in industrial, agricultural, and urban water consumption, environmental factors such as runoff have changed, and the environmental quality and ecosystem balance of the Lijiang River have been severely damaged [12,13]. Under the trend of global climate change, extreme climatic conditions are steadily increasing, and environmental changes pose new challenges to water environmental governance and protection [14,15,16]. It is of particular importance to know how to react to changing environmental conditions. Many water quality monitoring stations are distributed in the LRB and have obtained extensive long-term monitoring data. The important information contained in these data is needed to ensure adequate water quality management in the LRB.

Over the decades, studies have been undertaken on the correlation between water quality and environmental factors in watersheds. Land use change has been a hot topic, and considerable research on the impact of land use change on water quality changes has been conducted through regression analysis and model construction [17,18,19]. However, land use changes are mainly reflected on the land, which indirectly affects the pollutant content of rivers. To evaluate conventional water environmental factors in water bodies, water quality evaluation methods were used to investigate the impact of environmental factors on water quality indicators and microbial indicators for specific aquaculture ponds, lakes, and reservoirs [20,21,22]. These studies focused mainly on local areas or unique factors, and the results and scope have some limitations. The impact of environmental factors on the endogenous release of sediment has also attracted much attention [23,24], but these research findings are regional and primarily qualitative. Rivers often cross a vast region, and coastal topography, environment, climate, and other conditions can vary considerably along the course of the river. Different environmental factors often impact different water quality indicators across different regions. As a karst area, the nonpoint source (NPS) pollution on the surface of the LRB enters the underground river through sinkholes, and further diffuses into the surface water. The diffusion process and diffusion speed of the pollution are different from those in the basin with ordinary terrain. The section from Guilin to Yangshuo is a touristic hotspot, and pollution sources are also greater than those of general watersheds of the same scale. Statistical analysis methods are also very effective in water quality analysis. The water quality index (WQI) is widely used as an indicator to evaluate the quality of the water environment [25,26,27], but many factors influence water bodies, and the correlations are complicated. Wei et al. [28] analyzed water quality by canonical correlation analysis (CCA) and hierarchical cluster analysis (HCA), revealing the correlation between heavy metals and pollution indicators. Xiao et al. [29] used principal component analysis (PCA) and WQI to study the characteristics, water quality, and health risk assessment of trace elements in the water of the Loess Plateau. A single evaluation indicator can only provide a reference for pollution control and cannot provide targeted response measures to changes in certain environmental conditions. Reliable and accurate data on environmental concentrations is crucial for risk assessment [30].

The Lijiang River flows through Guilin city, agricultural lands, and karst areas. Thus, there is spatial heterogeneity in water quality indicators and environmental factors. Currently, systematic research on the temporal and spatial changes in water quality and the sensitivity of environmental factors in the Lijiang River is limited. Some water pollution control measures carried out in the LRB lack corresponding data support. Therefore, the objectives of this study were to: (1) expound on the temporal and spatial change characteristics and evolution trends of water quality indicators; (2) analyze the correlation between water quality indicators and environmental factors; (3) quantitatively assess the sensitivity and contribution rate of water quality indicators to environmental factors; (4) verify the feasibility of the statistical method; and (5) provide technical support and a theoretical basis for water environmental protection, water pollution control, and emergency treatment of environmental changes in the LRB.

## 2. Methodology

### 2.1. Study Area

**Figure 1:**
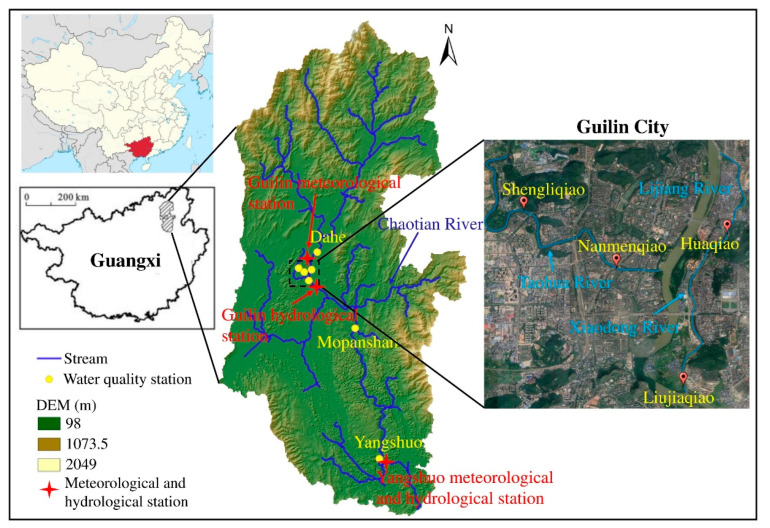
Study area and distribution of meteorological, hydrological, and water environmental stations.

The LRB originated at the eastern foot of Maoer Mountain, Xing’an County, Guilin city, Guangxi Province. The river is part of the Pearl River system, with a total length of approximately 214 km and a drainage area of approximately 5800 km^2^, as portrayed in Figure 1. The Lijiang River is a karst surface river that is characterized by thick and pure carbonate strata [12]; the topography of the Lijiang River from its source to the estuary can be divided into five geomorphological units: Zhongshan Mountain Landform, Hilly Valley Landform, Karst Peak Forest Plain Landform, Karst Peak Cluster Depression Landform, and Low Mountain and Hilly Landform. The riverbed of the Lijiang River primarily consists of a layer of sand and pebble over limestone with a thickness of 20~30 m [3], and the sediment consists of gravel and cobble with a median sediment size of 0.03~0.06 m [5]. The minimum temperature, maximum temperature, and annual average temperature in the LRB are −4.0 °C, 39.0 °C, and 18.8 °C, respectively. The north wind dominates year-round. As a rain-source river, the LRB is characterized by runoff heavily impacted by precipitation. The average precipitation in the basin is 1900 mm, and the average evaporation is 1485 mm. The rainy season is from late February to mid-August, and the relative humidity reaches 92%.

### 2.2. Data Sources

The meteorological data used in this study are from the National Meteorological Science Data Center (http://data.cma.cn/), and the hydrological data are from the Guilin Hydrological Bureau. The monitoring stations are located in Guilin and Yangshuo (Figure 1), and these stations collected data on daily maximum temperature, minimum temperature, precipitation, sunshine duration, wind speed, humidity, streamflow, and other indicators on the daily scale. Water environment data were obtained using both manual and automatic monitoring. The stations are displayed in Table 1 and are located on the Lijiang River and two tributaries, the Xiaodong River and the Taohua River. Among the stations, the Dahe and Mopanshan stations are located in the upper and lower reaches of Guilin city, respectively. Monitoring indicators include environmental factors (water temperature, pH, and dissolved oxygen (DO)) and water quality indicators (ammonia nitrogen (NH_4_-N), total phosphorus (TP), biochemical oxygen demand (BOD_5_), and chemical oxygen demand (COD_Mn_)) with a monthly data scale. Due to the different data scales, the meteorological and hydrological data are downscaled in this study, and the calculated monthly data are daily averages.

### 2.3. Analysis Method

#### 2.3.1. Trend Analysis

To analyze the temporal and spatial evolution of environmental factors and water quality indicators in the study area, we used the unary linear regression trend method to analyze the change trend and intensity on the time scale of the data from different monitoring stations in the LRB. The slope of the regression equation reflects the changing trend of the environmental factors and water quality indicators. A slope above 0 indicates that the index is rising in the time range, and a slope less than 0 indicates a downward trend. The magnitude of the slope can reflect the magnitude of the increase or decrease in the index. The calculation formula is as follows:

(1) $$\theta =\frac{n\times \sum_{i=1}^{n}\left(i\times C\right)-\left(\sum_{i=1}^{n}i\right)\left(\sum_{i=1}^{n}C\right)}{n\times \sum_{i=1}^{n}i^{2}-{\left(\sum_{i=1}^{n}i\right)}^{2}}$$

where *θ* is the changing trend, *i* is the time (month), *n* is the study period, and *C* is the concentration of the environmental factor at time *i*.

#### 2.3.2. Correlation Analysis

Environmental factors impact water quality indicators differently. In this study, correlation analysis methods are used to quantify the correlation between two indicators. Due to the poor regularity of the measured data and occasional outliers, the Spearman rank correlation coefficient method was adopted. For the original data with a sample size of *n*, the correlation coefficient *ρ* is:

(2) $$\rho =\frac{\sum_{i}\left(x_{i}-\bar{x}\right)\left(y_{i}-\bar{y}\right)}{\sqrt{\sum_{i}{\left(x_{i}-\bar{x}\right)}^{2}\sum_{i}{\left(y_{i}-\bar{y}\right)}^{2}}}$$

where *x_i_* and *y_i_* are the average of the descending positions of each raw data point.

#### 2.3.3. Sensitivity of Water Quality Indicators to Environmental Factors

Sensitivity can quantitatively reflect the impact of relative changes in environmental factors on the relative changes in the concentration of water quality indicators. This study uses the sensitivity coefficient defined by McCuen [31] to analyze the sensitivity of different water quality indicators in the LRB to environmental factors, calculated as follows:

(3) $$S\left(E\right)=\underset{∆E\to 0}{\lim}\left(\frac{∆WQ/WQ}{∆E/E}\right)=\frac{\partial WQ}{\partial E}\cdot \frac{E}{WQ}$$

where *S*(*E*) is the sensitivity coefficient of water quality indicators to environmental factors. The higher the absolute value of the sensitivity coefficient is, the greater the impact of relative changes in environmental factors on relative changes in water quality indicators. *WQ* and *E* represent water quality indicators and environmental factors, respectively. The advantage of the sensitivity coefficient is that it is dimensionless and can be compared among various dimensions.

#### 2.3.4. Contribution Rate

The contribution rate of environmental factors is obtained by multiplying the sensitivity coefficient and the relative change rate of the indicator over the years, and the calculation formula is as follows:

(4) $$\{\begin{array}{c} Con_{E}=S\left(E\right)\cdot RC_{E}\\ RC_{E}=\frac{n\times a_{E}}{\left|\bar{E}\right|}\times 100\% \end{array}$$

where *Con_E_* is the contribution rate of environmental factor *E* to the change in water quality index *WQ*, *RC_E_* is the multi-year relative change rate of environmental factors, *n* is the number of months, *a_E_* is the changing trend of environmental factors, and $\bar{E}$ is the monthly average value of environmental factors.

All statistical analyses and graphing were performed in R (Version 3.6.1, Lucent Technologies, Murray Hill, NJ, USA) [32] and OriginPro software (Version 2017, OriginLab Corporation, Northampton, MA, USA).

## 3. Results and Discussion

### 3.1. Meteorological and Hydrological Change Characteristics

**Figure 2:**
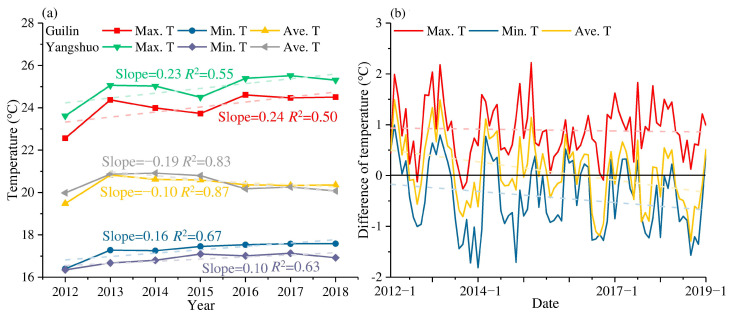
Characteristics of air temperature changes in Guilin and Yangshuo stations, (**a**) Air temperature, and (**b**) Temperature difference between Guilin and Yangshuo.

The hydrological and meteorological stations were located in Guilin and Yangshuo in the middle and lower reaches of the basin. The annual average temperatures of the two stations were between 19 and 21 °C and have exhibited a slight year-by-year downward trend since 2013 (Figure 2a). However, both the average daily maximum temperature and the minimum temperature increased. Maximum temperatures have exhibited a significantly increased rate, reaching 0.24 °C year^−1^ (Guilin) and 0.23 °C year^−1^ (Yangshuo), while the rates of minimum temperature were only 0.16 °C year^−1^ (Guilin) and 0.10 °C year^−1^ (Yangshuo). Guilin’s daily maximum temperature and minimum temperature have relatively slight fluctuations; they are 0.9 °C higher and 0.4 °C lower than the maximum temperature and minimum temperature in Yangshuo, respectively. Yangshuo is located in the lower reaches of the Lijiang River and has a small population, while Guilin has a slight temperature difference between day and night due to the heat island effect. There were also differences in the maximum temperature, the minimum temperature, and the average temperature between Guilin and Yangshuo, as indicated in Figure 2b. The maximum temperature in Yangshuo is on average approximately 1 °C higher than that in Guilin, while the gap between the minimum temperature increases year by year. Against the background of global warming and the increasingly extreme weather situations, the gap in the minimum temperature between Yangshuo (the location farther south) and Guilin is gradually widening, which has caused the average temperature to gradually change from being higher in Yangshuo to being higher in Guilin.

**Figure 3:**
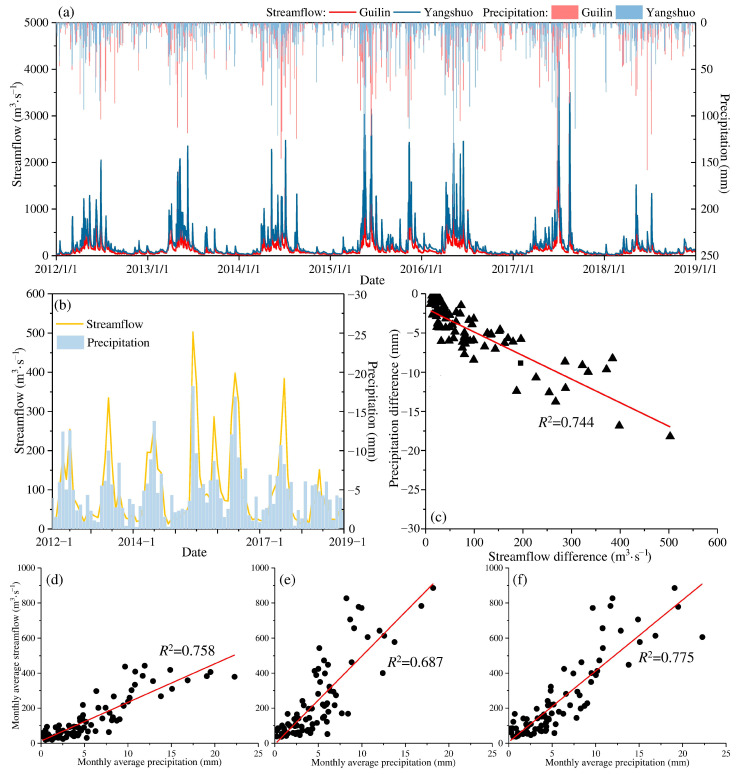
Characteristics of streamflow and precipitation changes in Guilin and Yangshuo stations, (**a**) Streamflow and precipitation, (**b**) Difference between Yangshuo and Guilin, (**c**) Correlation between difference of streamflow and precipitation, (**d**) Correlation between streamflow and precipitation at Guilin, (**e**) Correlation between streamflow and precipitation at Yangshuo, and (**f**) Correlation between streamflow at Yangshuo and precipitation at Guilin.

The streamflow in Yangshuo far exceeds that of Guilin because of the confluence of tributaries such as the Chaotian River. Due to the influence of rain, the maximum daily streamflow is more than 3000 m^3^·s^−1^ (Figure 3a), and the average monthly streamflow in Yangshuo is between 37 and 771 m^3^·s^−1^, over 1.7 times that in Guilin. The maximum streamflow in Guilin was measured at 3290 m^3^·s^−1^ on 15 August 2017, and still reached 2170 m^3^·s^−1^ (third peak) on the next day. In comparison, the maximum streamflow in Yangshuo measured 4660 m^3^·s^−1^ and 4030 m^3^·s^−1^ on 2 July and 3 July 2017, which were the highest and second peaks of streamflow at Yangshuo, respectively. On the peak day in Guilin (15 to 16 August 2017), Yangshuo measured the fourth peak of 3390 m^3^·s^−1^ and the third peak of 3500 m^3^·s^−1^, which were even higher than the maximum streamflow in Guilin.

As the main factor for the increase in streamflow, on 14 and 15 August 2017, the precipitation in Guilin reached 86 mm and 71 mm, respectively, while the corresponding amounts were only 33 mm and 28 mm in Yangshuo. In contrast to the streamflow, the precipitation in Guilin is generally greater than that in Yangshuo, and not just during heavy rain. Comparing the streamflow difference and precipitation difference between the two stations (Figure 3b,c), there is a significant negative correlation between the differences (*R*^2^ = 0.744), indicating that the excess precipitation in upstream Guilin will affect the streamflow in downstream Yangshuo in the same proportion. Based on this correlation, we analyzed the correlation between precipitation and streamflow at the same station and the correlation between Guilin precipitation and Yangshuo streamflow (Figure 3c–e). The *R*^2^ between precipitation and streamflow reached 0.758 in Guilin and 0.687 in Yangshuo, while the *R*^2^ of Guilin precipitation and Yangshuo streamflow (0.775) was even greater, which indicated that the precipitation feedback has a certain lag. Upstream precipitation often impacts downstream streamflow to a greater extent [33,34]. Therefore, although the precipitation at Guilin is relatively larger, it is reflected in the downstream flow due to the limitation of the width of the river channel. In addition, the greater the local streamflow is, the the lower the influence from upstream precipitation than from local precipitation.

### 3.2. Differences in Environmental Factors

**Figure 4:**
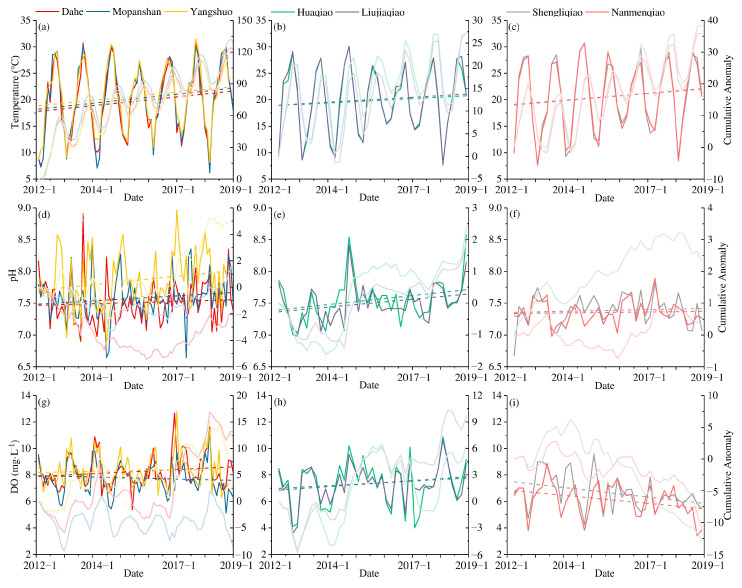
Changes in various environmental factors of different stations in the LRB, (**a**) Water temperature of Lijiang River, (**b**) Water temperature of Xiaodong River, (**c**) Water temperature of Taohua River, (**d**) pH of Lijiang River, (**e**) pH of Xiaodong River, (**f**) pH of Taohua River, (**g**) DO of Lijiang River, (**h**) DO of Xiaodong River, and (**i**) DO of Taohua River.

As a critical factor influencing changes in water quality, environmental factors also reflect differences in different regions of the LRB (Figure 4). The air temperature is the primary determinant of water temperature. At Dahe station, the average air temperature was 1 °C higher than the water temperature, and *R*^2^ between two temperature indexes was 0.86. The water temperature at each station is gradually rising, and the rising trend of water temperature at the stations of the same river is similar. The water temperature of the Lijiang River gradually rises from upstream to downstream (from north to south), and the rate of increase in water temperature is the largest (compared to two tributaries: Taohua River and Xiaodong River) among the watersheds with values between 0.043 and 0.048 °C month^−1^. Based on the changes in cumulative anomalies, it can be deduced that Mopanshan experienced the largest increase in water temperature. The two tributaries are located in the urban area of Guilin, and the Xiaodong River is similar to Mopanshan, whereas the Taohua River fluctuates over a relatively large range. The growth rates of the Xiaodong River are the lowest, at 0.023 and 0.026 °C month^−1^, respectively, and the growth rates of the Taohua River are 0.036 and 0.039 °C month^−1^.

The pH of the water body can reflect the water chemistry characteristics of the water body as a whole, and it is one of the important indicators with which to evaluate water quality. The pH value of the LRB was generally between 7.0 and 8.5, which was weakly alkaline with upward trends. The pH of the two tributaries was relatively low compared to the Lijiang River; the Xiaodong River had the highest increase, while the pH of the Taohua River was relatively stable. The entire section of the Xiaodong River is located in the urban area of Guilin. The water catchment area was primarily on the east bank of the Lijiang River, but the pH value maintained an upward trend, which is different from the pattern observed for the Taohua River located on the west bank. In addition to point source pollution, the surface pollutants on the east bank of the Lijiang River in Guilin city are mainly alkaline substances, such as agricultural NPS pollution. The pH value of the Lijiang River gradually increases from upstream to downstream; Yangshuo has a significantly higher pH than the rest of the stations, and cumulative anomalies remain positive and rise steadily. Karst features and woodlands dominate the riparian catchment area of the Mopanshan to Yangshuo section, which indicates that the overall pH of the woodland soil in this section is alkaline or weakly alkaline, and alkaline substances are transported to the water body via surface runoff during the rainfall process.

The DO concentration in the Lijiang River is significantly higher than that in the tributaries, and over 75% of the DO exceeds 6.8 mg·L^−1^. The DO concentration of the Xiaodong River is slightly lower than that of the Lijiang River and is significantly higher than that of the Taohua River. Although the Xiaodong River is a tributary, both the upstream and downstream reaches connect with the Lijiang River, so the Huaqiao station upstream of the Xiaodong River also has large fluctuations. The pH values of the Xiaodong River and Dahe stations have similar growth trends, and the change rates are between 0.009 and 0.013 mg·L^−1^·month^−1^. The Dahe and Mopanshan stations are located in the upper and lower reaches of the Guilin urban area, respectively, and demonstrate significant differences. Mopanshan’s DO demonstrated a downward trend, indicating that the water quality deteriorates to a certain extent while flowing through the Guilin urban area. As a tributary of the Lijiang River in Guilin, the Taohua River has a low water volume, a low flow rate, and a relatively low DO concentration relative to those of the mainstream. However, the DO concentration in the Taohua River continues to decrease year by year, which implies that the Taohua River carries a large amount of oxygen-consuming substances, which deteriorates the water quality of the Lijiang River to some extent. With the exception of lower-quality tributaries, the discharge of a large quantity of wastewater from the WWTP increased the DO consumption. The sections from Mopanshan to Yangshuo are all relatively unpopulated mountainous areas, and the external source is only surface runoff, so the concentration of DO gradually rises. The streamflow of the Lijiang River gradually increased from Dahe to Yangshuo. In addition to the input of surface runoff, the inflow of tributaries from the Taohua River, the Qifeng River, and the Chaotian River also increased the volume of water in the river.

### 3.3. Evolution Trend of Water Quality Indicators

**Figure 5:**
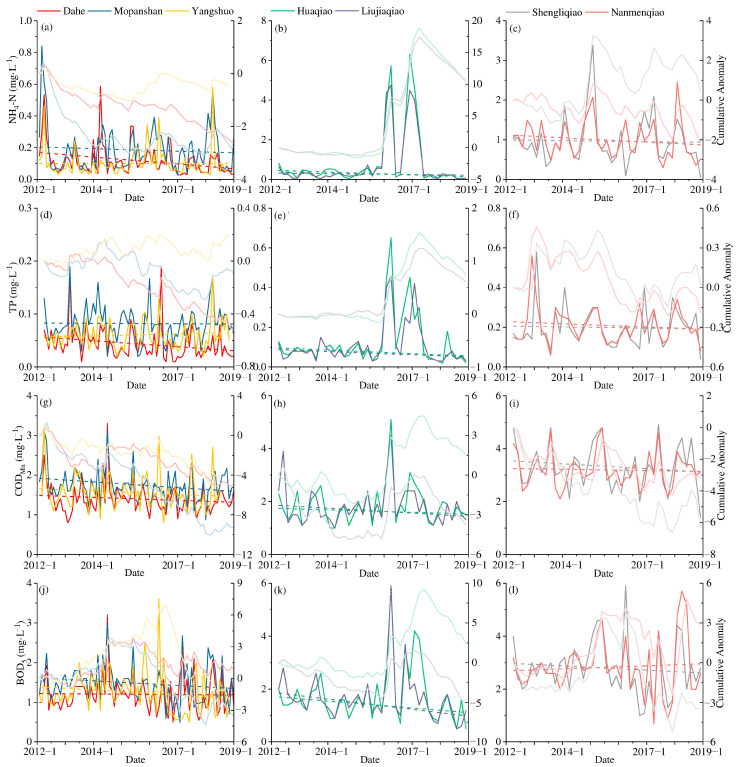
Changes in various water quality indicators at different stations in the LRB, (**a**) NH_4_-N of Lijiang River, (**b**) NH_4_-N of Xiaodong River, (**c**) NH_4_-N of Taohua River, (**d**) TP of Lijiang River, (**e**) TP of Xiaodong River, (**f**) TP of Taohua River, (**g**) COD_Mn_ of Lijiang River, (**h**) COD_Mn_ of Xiaodong River, (**i**) COD_Mn_ of Taohua River, (**j**) BOD_5_ of Lijiang River, (**k**) BOD_5_ of Xiaodong River, and (**l**) BOD_5_ of Taohua River.

The changing trends of the four water quality indicators NH_4_-N, TP, COD_Mn_, and BOD_5_ at seven stations in the study area are portrayed in Figure 5. Overall, the water quality of the Lijiang River is substantially better than that of the tributaries. The declining trend of NH_4_-N in Dahe is the most obvious, but Mopanshan has the largest decline in the cumulative anomaly value. Yangshuo, the most downstream station in the study area, has undergone a minimal change. The concentration of NH_4_-N increases in the section passing through Guilin city and gradually drops to a lower level in the rural area, with a trend opposite that of the concentration of DO. Similarly, the TP in Dahe also indicated a strong downwards trend, followed by Mopanshan. However, there was an upward trend in Yangshuo, which indicated that the water quality in the upper reaches of Guilin has been improving year by year, and the pollution level increases slightly as the river flows through the urban area of Guilin, but it still remains within the range of self-purification capacity. The influx of tributaries such as the Qifeng River and Chaotian River in the lower reaches of Guilin City also brings a certain amount of nitrogen and phosphorus. These tributaries have relatively low streamflow, flow through villages and farmlands, and receive a certain amount of agricultural NPS nitrogen. In the section from Mopanshan to Yangshuo, there are dozens of villages and farmlands on both sides of the Lijiang River, and agricultural NPS pollution has become the primary source of nitrogen and phosphorus in this section. COD_Mn_ is the optimal pollution control indicator for the Lijiang River, and the concentration demonstrates a significant declining trend year by year and is controlled at levels below 2 mg·L^−1^. BOD_5_ is the oxygen consumption of aerobic microorganisms in the water body to decompose organic matter into the inorganic matter under certain temperature conditions within 5 days, and it can indirectly reflect the relative content of organic matter in the water. Although the BOD_5_ maintained a downward trend, the cumulative anomaly values demonstrated an upward trend and remained positive for a certain period. The cumulative anomaly value in Yangshuo was the highest, indicating that the pollutants brought from Mopanshan to Yangshuo contain much organic matter.

The NH_4_-N concentration in the Xiaodong River can be maintained at a level below 1.0 mg·L^−1^ (Level III of environmental quality standards for surface water of China, GB 3838-2002) most of the time. However, from the beginning of 2016 to the first half of 2017, there were two surges in NH_4_-N concentration. The peak value of NH_4_-N at the Huaqiao station reached 6.30 mg·L^−1^. The proportion of NH_4_-N in TN is often less than 0.1 in water bodies with a higher DO concentration, while during the period of the NH_4_-N outbreak, the proportion increased to over 0.5 and reached 0.89, indicating that the two abnormal results were mainly derived from the sudden input of external NH_4_-N rather than internal nitrogen conversion. Additionally, the input of NH_4_-N consumes a large amount of DO, and the DO concentration of the Huaqiao station dropped from above 7 mg·L^−1^ to below 5 mg·L^−1^. The pollution sharply increased as a result of two accidents and returned to normal levels within a short period. Due to the low streamflow of the Xiaodong River, there was no significant impact on the Lijiang River. Around these two peaks, the concentration of NH_4_-N in the Xiaodong River changed from an upward trend to a downward trend. Like nitrogen, the TP concentration of the Xiaodong River also increased twice in 2016 due to external sources. Although the nitrogen and phosphorus in the Xiaodong River have conditions of abnormal pollution concentrations, the overall trend has continued to decline. COD_Mn_ and BOD_5_ were also affected by the unexpected situation, and the cumulative anomaly value increased significantly. However, in the four indicators, the concentration of Huaqiao is higher than that of Liujiaqiao, scilicet the Xiaodong River gradually undergoes a self-purification process during the flow process.

The concentration of NH_4_-N in the Taohua River was relatively high, and it was maintained at the level of 1.5 mg·L^−1^, with a slight downward trend from 2012 to 2018. The TP of the Taohua River is primarily kept at the level of 0.3 mg·L^−1^. Shengliqiao station fluctuates more frequently than other stations, caused by the sewage discharged from the WWTP located upstream of the Taohua River. Water quality from the outlet of the WWTP to the confluence of the Taohua River and the Lijiang River gradually improved, the organic matter content also gradually decreased, and the BOD_5_ index gradually decreased.

### 3.4. Correlation Analysis of Environmental Factors and Water Quality Concentration

**Figure 6:**
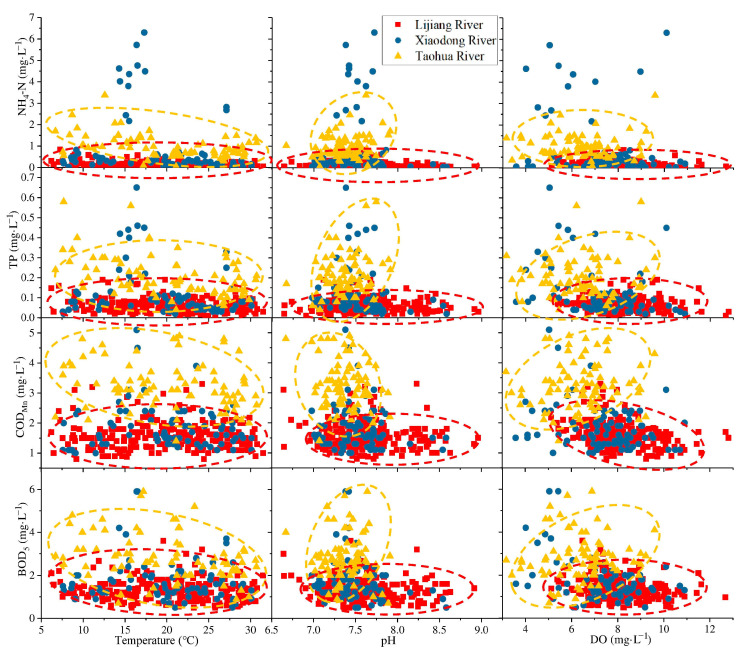
Differences in the impact of different river environmental factors on water quality indicators.

Environmental factors play an essential role in the migration and transformation of pollutants in water bodies, thereby affecting the concentration of pollutants and the quality of the aquatic environment. Different environmental factors impact different water quality indicators, and the same indicator also differs across different rivers and regions. Figure 6 uses environmental factors (water temperature, pH, and DO) as the abscissa, and water quality indicators (NH_4_-N, TP, COD_Mn_, and BOD_5_) as the ordinate, indicating the distribution of water quality indicators for different environmental factors in different rivers. Although there are several stations in the same river, there is little difference among them, while the differences between the different rivers are significant.

The Xiaodong River is a tributary whose upper and lower sections are connected to the Lijiang River. While streamflow is low and occasional outliers occur, most results are similar to the distribution of the Lijiang River. Under the same environmental conditions, almost all water quality indicators of the Taohua River are higher than those of the Lijiang River. Due to the large streamflow, the distribution of the Lijiang River water quality indicators is relatively stable with changes in environmental factors. Overall, the COD_Mn_ indicates a significant downward and upward trend when the DO and streamflow increase. Several water quality indicators in the Taohua River also tend to change depending on environmental factors, which illustrates that the nature of the river itself strongly influences the impact of environmental factors on water quality indicators. In contrast, the effects of various locations on the same river are more similar.

Based on this, this study conducts a statistical analysis of the results of different stations. Table 2 exhibits the Spearman correlation coefficients between environmental factors and water quality indicators at different stations. Due to the absence of streamflow data for tributaries, the correlation coefficient between streamflow and water quality indicators is only calculated for the Lijiang River. Not all indicators are statistically correlated.

Nitrogen and phosphorus in water bodies are primarily derived from external sources. The Taohua River flows through agricultural land before it merges into the Lijiang River and has a low flow, making it susceptible to the seasonal inputs of fertilizer from agricultural work. However, runoff from the LRB is high in summer and low in winter, and water flow and temperature are often accompanied by changes. Tributaries are more significantly affected, with sediment resuspension at the bottom of the river due to flow changes releasing a certain amount of phosphorus to flow downstream. TP in the Xiaodong River is weakly and negatively correlated with DO (0.2 < *ρ* < 0.4), which indicates that the input of organic oxygen-depleting substances often accompanies the increase in TP in the Xiaodong River. TP of the Lijiang River is relatively stable and is less affected by environmental factors, and they all have a very weak correlation (*ρ* < 0.2) if they are correlated.

In both the Lijiang River and its two tributaries, NH_4_-N negatively correlates with water temperature, and rising temperatures may promote nitrification. As heat and rain are both higher during the same season, the increase in water flow and temperature increased DO by diluting NH_4_-N, resulting in a decrease in NH_4_-N concentration. The dilution of NH_4_-N caused by flow changes is the primary reason for the correlation between water temperature and NH_4_-N.

### 3.5. Sensitivity of Water Quality Indicators to Environmental Factors

The sensitivity coefficients of water quality indicators to various environmental factors are calculated (Table 3), and most of them are highly sensitive. The pH value is extremely sensitive to NH_4_-N, and in the results from each station, the |*S*(*E*)| values are nearly all above 1. While the pH value is moderately sensitive to TP, at a single station, Huaqiao, the sensitivity of TP to the pH value was −8.87. A single station may demonstrate abnormal results due to various external factors. However, changes in phosphorus in water bodies are mainly from external sources, which are often accompanied by rainfall, which is highly variable. The fluctuation in pH value is low when the rainfall changes, leading to high sensitivity of TP to the pH value.

Except for BOD_5_ in Yangshuo, the sensitivity of all water quality indicators to water temperature was negative (−0.916~−0.143). The absolute value of TP sensitivity is between 0.5 and 1, which means that for every 1 °C increase in water temperature, the concentration of the water quality indicator will decrease by 0.5~1 mg·L^−1^. The water quality indicators are always below 1 mg·L^−1^ throughout the year, indicating that other environmental factors have a tremendously positive influence on the water quality indicator concentration. Temperature can accelerate the oxidation of NH_4_-N and organic matter because of the high concentration of DO in water, resulting in negative sensitivity.

DO may react with various water quality indicators to weaken its concentration. Therefore, it is mainly reflected as a negative correlation. However, there are specific differences at different stations. Shengliqiao and Nanmenqiao are located at the upstream and downstream reaches of the same tributary, respectively. The absolute values of the sensitivity of TP to DO are approximately 2, but the sensitivity of these variables is the opposite. This result is primarily caused by the fact that there are WWTPs, farmland, and other external sources located in the vicinity upstream of Shengliqiao, and a large amount of phosphorus is deposited in the sediments. When the DO concentration increases with the disturbance of the water body, the phosphorus in the sediment that enters the water with the resuspended particles reacts with DO and dissolves in the water body, resulting in increased TP concentration. Nanmenqiao is located at the confluence of two rivers, the water body is disturbed and flooded, the phosphorus content in the sediment is low, and DO can inhibit the release of phosphorus in the sediment and reduce the TP concentration of the water body.

The sensitivity of the various water quality indicators to environmental factors varies considerably. The four water quality indicators all have negative sensitivity to water temperature, pH, and DO. The absolute value of the mean value of the sensitivity coefficient of NH_4_-N to each environmental factor, in order from large to small, was pH, DO, and water temperature; that of TP was in the order DO, water temperature, pH; that of COD_Mn_ was in the order DO, pH, water temperature; that of BOD_5_ was in the order pH, DO, water temperature.

### 3.6. Water Quality Contribution Rate

Based on calculations of the sensitivity of water quality indicators to environmental factors and estimates of each environmental factor’s contribution to changes in water quality indicators (Table 4), the contribution rates are all negative. Water temperature has contribution rates of −12% to NH_4_-N at Mopanshan and −9% to TP at Dahe. Water temperature changes considerably throughout the year and therefore has some periodic impact on the water quality indicators. The contribution rate of water temperature to TP was not the highest among all these environmental factors, and the contribution rates of DO-to-TP and pH-to-TP were over −6%. COD_Mn_ is directly related to DO, and the changes in water temperature influence the COD_Mn_ through DO; thus, DO exhibits the highest contribution rate. Increasing the DO content can improve water quality and reduce the organic matter content of the water body, significantly impacting the reduction in BOD_5_.

To explore the contribution of environmental factors to water quality indicators at different stations, the environmental factor with the largest contribution rate to water quality at each station is defined as the dominant factor, as indicated in Table 5. The dominant factor of most stations on the Lijiang River is the water temperature, while DO primarily dominates for the Taohua River. The four stations of the tributaries all use DO as the dominant factor of NH_4_-N, reaching −17%, −11%, 15%, and 13%, respectively. In the Lijiang River with higher streamflow, dominant factors are represented by water temperature and pH value. DO can directly react with NH_4_-N to affect its concentration. However, due to the large streamflow in the Lijiang River, the DO concentration is relatively high, and the NH_4_-N concentration is relatively low; the influence of DO is weakened, and other indicators may indicate their influence. DO and pH are the primary factors influencing endogenous phosphorus release [35,36], and their contribution to TP varies among regions. TP in the Lijiang River is mainly affected by pH and water temperature, indicating that changes in phosphorus in the Lijiang River are primarily caused by the combination with metal ions and decomposed by microorganisms and organic matter. In contrast, phosphorus is primarily decomposed by organic matter in the tributaries. Both COD_Mn_ and BOD_5_ are indicators that express water quality through chemical reactions and oxygen are parts of the reaction. Consequently, the dominant factors are essentially water temperature and DO. The DO concentration in the Lijiang River is relatively stable, with water temperature being the major contributing factor. In contrast, in tributaries, DO concentrations are low and vary widely, and the contribution rate of DO is higher than that of water temperature.

### 3.7. Spatial Difference and Water Quality Management

**Figure 7:**
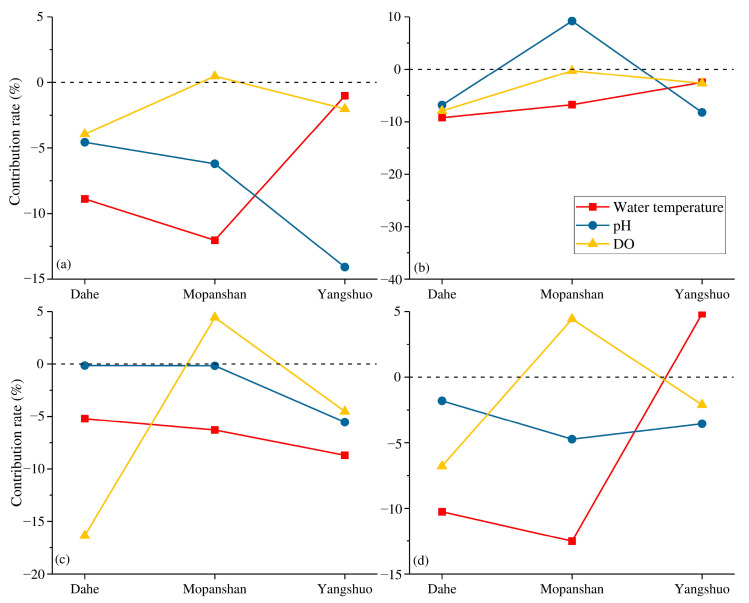
Changes in the contribution rate of environmental factors to water quality indicators in the Lijiang River, (**a**) NH_4_-N, (**b**) TP, (**c**) COD_Mn_, and (**d**) BOD_5_.

The pollution contribution rate reflects the overall results of a broad area. There are specific differences among stations, and these differences will impact water pollution control actions. The changes in the contribution rate of environmental factors to water quality indicators of the Lijiang River are portrayed in Figure 7. The Dahe to Mopanshan section and the Mopanshan to Yangshuo section have the opposite effects; the Lijiang River flows through Guilin city in the Dahe to Mopanshan section and receives the Taohua River, a tributary that flows through the urban area. In contrast, the Mopanshan to Yangshuo section is dominated by agricultural land and mountainous areas. Differences in topography and population have led to changes in the impact of environmental changes on water quality indicators.

NH_4_-N, TP, COD_Mn_, and BOD_5_ are indicators for which lower values indicate better water quality. The DO in Mopanshan has a positive contribution to NH_4_-N, COD_Mn_, and BOD_5_, which means that an increase in DO will promote an increase in these three indicators. With the exception of the contribution rate of DO to NH_4_-N, which was only 0.47%, the other two indicators exceeded 4%. DO will clearly inhibit the growth of these indicators, as evidenced by Dahe and Yangshuo. However, DO in Mopanshan has a negative contribution to TP, and the contribution level is also significantly lower than that of Dahe. This result is primarily due to external pollution. First, the wastewater discharged from WWTPs in Guilin city has higher pollution concentrations [37], so pollutants increase as the Lijiang River flows through Guilin city. However, the confluence with the tributary Chaotian River increases the streamflow, promotes the exchange of water bodies, and increases the DO. However, the inflow of the Chaotian River could not eliminate the deterioration of water quality caused by Guilin city. The contents of DO and pollutants increased simultaneously, resulting in the positive contribution of DO to pollutants. In other words, the conclusion obtained only from the perspective of data analysis may be inaccurate or the opposite, and the correlation between DO and pollutants needs to be interpreted with caution. Different results from different distributions can be interpreted and excluded. Although the section from Mopanshan to Yangshuo flows through farmland and livestock areas, the contribution of DO to water quality tends to be positive, indicating that rainfall causes pollutants carried by surface runoff into the water body to be limited.

The contribution rates of water temperature to these indicators are negative, and the water quality can be improved by increased temperatures, while the BOD_5_ of Yangshuo increases with the increase in water temperature. In the section from Dahe to Mopanshan, the contribution trends of water temperature to NH_4_-N, COD_Mn_, and BOD_5_ are opposite that of DO and are the same as that of the pH value. The DO content increases in summer, and the DO in the river water that flows through Guilin city interacts with and leads to reductions in pollution, NH_4_-N, and inorganic and organic pollutants. The removal efficiency of TP is relatively weak because TP does not interact with DO. In the section from Mopanshan to Yangshuo, the contribution rate of rising water temperature to NH_4_-N and TP drops sharply, and BOD_5_ increases with increasing temperature. In this section, the input of an external nitrogen source is relatively low, increasing the water temperature can effectively reduce the nitrogen content of the water body, and the external pollution of organic matter, NH_4_-N, and TP are more serious.

From the changes in the contribution rate of different regional environmental factors to water quality, it can be observed that external sources affect not only the concentration of various water quality indicators but also the sensitivity of water quality indicators to the external environment. The exogenous pollution in Guilin city is primarily domestic and industrial wastewater produced by the resident population, while it is mainly organic matter and phosphorus in the Mopanshan to Yangshuo section. Phosphorus is derived primarily from agricultural fertilizers and organic matter from tourism. The section from Guilin city to Yangshuo is a major tourist route. According to statistical data, the annual tourist population of Guilin rose from 31.1 million in 2012 to 106.41 million in 2018. Many round-trip cruise ships have brought a large quantity of organic pollutants to the Lijiang River. Since the Lijiang River is the drinking water source for the entire basin and Guilin area, its water quality is closely related to the health and safety of residents. The water pollution control of the LRB faces a marked threat, whether through organic pollutants or agricultural NPS pollution.

Due to the backward development concept, the lack of overall management, and the vulnerability of the river ecosystem, the health of the river tourism ecosystem is threatened, which affects the sustainable development of the river tourism. Therefore, to preserve the sustainable development and water quality of the Lijiang River, a series of measures should be implemented, such as controlling pollution discharge, adopting better sewage treatment devices, limiting industrial wastewater and sewage discharge, building dams and wetlands in the lower reaches of the Lijiang River, and intercepting agricultural NPS pollution and particulates. Additionally, different water environment control methods were used in different areas, such as oxygenation or chemical means to increase pH in Dahe and Yangshuo to control water pollutants. The results of this study can provide data reference for the LRB and water pollution control. The correlation study of river pollutants has played a good data support and reference role for river health evaluation, and thus reflects the health status of the LRB.

This study also proves the feasibility of statistical methods (trend analysis, correlation analysis, sensitivity analysis, and contribution rate calculation), particularly the latter two methods. In previous studies, these methods commonly used in hydrological research (Table 6), can also be used to quantify the correlation of water environment indicators. These methods can also be extended to other basins, particularly areas with complex terrain. For terrains such as karst basins, the modeling process is complicated, and the accuracy is poor [38]. Various scenarios need to be set for the influence of different factors. Statistical analysis methods can obtain relevant correlations relatively quickly, thus providing assistance to the government and the department of environmental protection.

## 4. Conclusions

Studying the relevance of environmental factors and water quality revealed spatial differences in water pollution and responses to environmental factors in the LRB. Water quality deteriorates and the DO concentration decreases considerably when the river passes through Guilin city, and the Taohua River carries a large amount of oxygen-consuming substances to the Lijiang River. NPS pollution is the primary source of pollution from Mopanshan to Yangshuo. The nature of the river itself heavily influences the impact of environmental factors on water quality indicators, while the different locations on the same river vary less widely in their effects. Four water quality indicators were negatively correlated with water temperature, pH, and DO. The sensitivities of NH_4_-N and COD_Mn_ to streamflow increase with the flow direction, while the sensitivity of TP gradually decreases.

The pollution discharge in the urban area of Guilin led to the decline in water quality in the section from Guilin to Mopanshan. In contrast, the NPS pollution in the section from Mopanshan to Yangshuo deteriorated the water quality, but the effect was limited. External sources affect the concentrations of various water quality indicators and the sensitivity of water quality indicators to the external environment. Phosphorus pollutants from Mopanshan to Yangshuo are mainly from agricultural fertilizers and organic matter from tourism. A series of measures should be implemented to control pollution and use different water environment control methods in different areas, such as using oxygenation or chemical means to increase pH in Dahe and Yangshuo to control water pollutants. Tourism and special karst topography make LRB’s calculation results unique, but the research method can be extended to other watersheds. We will continue to collect data and build the model based on machine learning methods in the near future.

## Figures and Tables

**Table 1:** Information on monitoring stations.

Type of Data	Indicators	Stations	Location	Note
Meteorological	Air temperature, precipitation, etc.	Guilin	110°18′ E, 25°19′ N	
		Yangshuo	110°30′ E, 24°46′ N	
Hydrological	Streamflow	Guilin	110°19′ E, 25°14′ N	
		Yangshuo	110°30′ E, 24°46′ N	
Water environment	Water temperature, DO, pH, BOD_5_, TP, etc.	Dahe	110°19′ E, 25°19′ N	Lijiang River
		Mopanshan	110°25′ E, 25°7′ N	Lijiang River
		Yangshuo	110°29′ E, 24°47′ N	Lijiang River
		Huaqiao	110°18′ E, 25°16′ N	Xiaodong River
		Liujiaqiao	110°17′ E, 25°15′ N	Xiaodong River
		Shengliqiao	110°16′ E, 25°17′ N	Taohua River
		Nanmenqiao	110°17′ E, 25°16′ N	Taohua River

**Table 2:** Spearman correlation coefficient of different river environmental factors and water quality indicators.

Indicators	River	Water Temperature	pH	DO
NH_4_-N	Lijiang River	−0.230 **	−0.209 **	−0.141 *
	Xiaodong River	−0.410 **	−0.113	−0.146
	Taohua River	−0.416 **	0.176	−0.038
TP	Lijiang River	−0.126	−0.054	−0.129 *
	Xiaodong River	−0.250 *	−0.303 **	−0.321 **
	Taohua River	−0.437 **	0.143	−0.084
COD_Mn_	Lijiang River	0.007	−0.051	−0.255 **
	Xiaodong River	−0.012	−0.032	−0.209
	Taohua River	−0.282 *	−0.089	0.120
BOD_5_	Lijiang River	−0.072	−0.168 **	−0.153 *
	Xiaodong River	−0.124	−0.211	−0.248 *
	Taohua River	−0.343 **	0.023	0.115

Note: “*”: significant at *p* < 0.05, “**”: significant at *p* < 0.01.

**Table 3:** Sensitivity coefficients of different water quality indicators to environmental factors.

	Temperature	pH	DO
NH_4_-N	−0.394 *	−1.226 **	−0.645 *
TP	−0.376 *	−0.162	−0.646 *
COD_Mn_	−0.143	−0.733 *	−0.871 *
BOD_5_	−0.326 *	−0.863 *	−0.340 *

Note: “*”: 0.2 < |*S*(*E*)| < 1, highly sensitive, “**”: |*S*(*E*)| > 1: extremely sensitive.

**Table 4:** The average contribution rate of different environmental factors to water quality indicators (%).

	Water Temperature	pH	DO
NH_4_-N	−6.09	−4.64	−0.66
TP	−5.61	−6.28	−6.27
COD_Mn_	−2.93	−2.36	−9.69
BOD_5_	−5.16	−3.31	−6.74

**Table 5:** Dominant factors of different monitoring stations.

	NH_4_-N	TP	COD_Mn_	BOD_5_
Dahe	Water temp.	Water temp.	DO	Water temp.
Mopanshan	Water temp.	pH	Water temp.	Water temp.
Yangshuo	pH	pH	Water temp.	Water temp.
Huaqiao	DO	pH	DO	pH
Liujiaqiao	DO	pH	DO	DO
Shengliqiao	DO	DO	DO	DO
Nanmenqiao	DO	DO	DO	DO

**Table 6:** Comparison of different works use similar methods.

Method	Description	Area	Reference
Sensitivity analysis and contribution	Sensitivity of vegetation to climate change, contribution of climatic factors to vegetation.	China	Jiao et al. (2021) [39]
Temporal trend, sensitivity analysis	Temporal trends in meteorological factors, sensitivity of Runoff to climate variability.	Haihe Basin	Wang D. et al. (2019) [40]
Sensitivity analysis, Mann-Kendall change point	Sensitivity of streamflow change, attribution analysis of streamflow change	Baiyangdian catchment	Wang H. et al. (2021) [41]
Trend analysis, sensitivity and contribution analysis	Trend, change point, sensitivity, and contribution in evapotranspiration, precipitation, and aridity.	Iran	Nouri and Bannayan (2019) [42]

## Data Availability

Not applicable.
